# Partially hydrolyzed guar gum attenuates symptoms and modulates the gut microbiota in a model of SARS-CoV-2 infection

**DOI:** 10.1017/gmb.2024.7

**Published:** 2025-01-14

**Authors:** Jiayue Yang, Isaiah Song, Misa Saito, Tenagy Hartanto, Takeshi Ichinohe, Shinji Fukuda

**Affiliations:** 1 Institute for Advanced Biosciences, Keio University, Tsuruoka, Yamagata, Japan; 2 Metagen, Inc., Tsuruoka, Yamagata, Japan; 3 Division of Viral Infection, Department of Infectious Disease Control, International Research Center for Infectious Diseases, Institute of Medical Science, The University of Tokyo, Tokyo, Japan; 4 Gut Environmental Design Group, Kanagawa Institute of Industrial Science and Technology, Kawasaki, Kanagawa, Japan; 5 Transborder Medical Research Center, University of Tsukuba, Tsukuba, Ibaraki, Japan; 6 Laboratory for Regenerative Microbiology, Juntendo University Graduate School of Medicine, Tokyo, Japan

**Keywords:** PHGG, COVID-19, SARS-CoV-2, gut microbiota, SCFAs, UDCA

## Abstract

The coronavirus disease 2019 (COVID-19) pandemic has caused health issues worldwide. Studies have suggested that modulation of the gut microbiota could attenuate the severity of COVID-19 symptoms. In light of this, we explored the effects of the prebiotic dietary fibre partially hydrolyzed guar gum (PHGG) on SARS-CoV-2 infection in a Syrian hamster model, hypothesizing that modulation of the gut microbiome and intestinal metabolites through PHGG administration would improve COVID-19 disease outcomes. Eight hamsters each were assigned to the PHGG administration and control groups. The PHGG group was given a diet supplemented with 5% PHGG for two weeks. Consequently, PHGG improved the host survival rate to 100% compared to 25% of the control group (P = 0.003) and attenuated morbid weight loss. Another non-infected set of hamsters was used for the analysis of the gut microbiome composition with 16S rRNA amplicon sequencing, serum, and faecal metabolites with GC–MS and LC–MS. PHGG altered the gut microbiome composition and increased the relative abundances of *Ileibacterium*, *Bifidobacterium*, and *Prevotella.* Furthermore, it elevated the concentrations of faecal valeric acid, propionic acid, ursodeoxycholic acid, and serum deoxycholic acid. Taken together, our data suggest that the prebiotic PHGG modulates gut metabolites and has the potential to reduce COVID-19 morbidity.

## Introduction

The coronavirus disease 2019 (COVID-19) pandemic is the source of major health concerns. While most cases are relatively mild, severe acute respiratory syndrome coronavirus 2 (SARS-CoV-2) infection-induced cytokine storms. Cytokine storms arise from dysregulation of immune responses, which can cause debilitating symptoms through systemic hyperinflammation and may result in death (Zhang et al., 2022a). Although several factors such as smoking history, diabetes, and hypertension are reported to be correlated with the severity of symptoms (Rahman and Sathi, 2021), serious disease outcomes remain difficult to predict and treatment options in these cases are limited. Furthermore, a report showed that 45% of COVID-19 survivors continued to suffer from a range of unresolved symptoms after four months (O’Mahoney et al., 2023). Despite the development of vaccines, new SARS-CoV-2 variants capable of escaping the adaptive immune response elicited by vaccination continue to emerge (Cao et al., 2022). Therefore, it is necessary to continue finding ways to treat and prevent the disease, particularly in attenuating the inflammation and cytokine storm characteristic of severe cases.

The gut microbiota is strongly connected with the host immune system. Gut microbes produce many kinds of metabolites such as short-chain fatty acids (SCFAs) and secondary bile acids, which have various biological functions such as host immune system modulation and anti-inflammation (Yang et al., 2020; Fan and Pedersen, 2021; Hu et al., 2021). Furthermore, the gut microbiota can regulate the local intestinal immune system and greatly influence systemic immune responses (Furusawa et al., 2013; Atarashi et al., 2015). Studies have shown that there is a crosstalk between the gut microbiota and the host pulmonary system, which is referred to as the gut-lung axis (Zhang et al., 2020). There are several reports of links between the gut microbiota and SARS-CoV-2 infection, in that the gut microbiome compositions of patients with COVID-19 show low diversity and fewer SCFA producers compared to healthy persons (Gaibani et al., 2021; Ren et al., 2021). A cohort study showed that there are associations between the composition of the gut microbiota and the severity of COVID-19, including elevated concentrations of inflammatory markers (Yeoh et al., 2021). Alterations in gut bacteria may be associated with excessive inflammatory responses in patients with severe COVID-19 symptoms (Yeoh et al., 2021). Recently, the SCFA acetic acid has been recognized for its ability to inactivate SARS-CoV-2 *in vitro* (Hikmet et al., 2020). Furthermore, it has been reported that the gut microbiota-derived secondary bile acid ursodeoxycholic acid (UDCA) is able to reduce the expression of viral host receptor angiotensin-converting enzyme 2 (ACE2) protein through protein-ligand interaction with farnesoid X receptors (FXR) (Brevini et al., 2023). Furthermore, UDCA treatment has been correlated with positive clinical outcomes in COVID-19 cases (Brevini et al., 2023). Therefore, we considered that the modulation of gut microbiome profile may contribute to the attenuation of the inflammation caused by SARS-CoV-2 infection.

Gut microbiome profiles are influenced by the daily diet of the host and studies have suggested that dietary interventions that modulate the gut microbiota might have the potential to improve clinical outcomes of COVID-19 (Hou et al., 2022; Wastyk et al., 2021; Merino et al., 2021). It is well-known that the gut microbiota produces SCFAs from dietary fibre (Fan and Pedersen, 2021). In a large prospective survey, a plant food-rich diet was shown to be associated with lower risk and severity of COVID-19 (Merino et al., 2021). Vegetarian patients have similarly been associated with decreased severity of COVID-19-related inflammation (Hou et al., 2022). Therefore, we considered that dietary intervention may contribute to the prevention of inflammation caused by SARS-CoV-2 infection.

In this study, we attempted to use the prebiotic dietary fibre partially hydrolyzed guar gum (PHGG) to modulate the gut microbiome and its metabolites for the prevention and attenuation of the inflammation caused by SARS-CoV-2 infection. PHGG is a water-soluble, low-viscosity dietary fibre (Yoon et al., 2008). It is manufactured by enzymatic hydrolysis of a highly viscous dietary fibre guar gum made from *Cyamopsis tetragonoloba*, also known as cluster beans or guar, which grows in tropical and subtropical regions and is widely cultivated in India, Pakistan, and the USA (Jaramillo et al., 2019). It is often used as a prebiotic food to improve constipation (Kapoor et al., 2017). Previous studies reported that PHGG is able to alter the gut microbiome by increasing the abundance of functional bacteria such as *Bifidobacterium*, and stimulating the intestinal SCFA concentrations (Okubo et al., 1994; Ohashi et al., 2015). Moreover, in a human clinical study, PHGG has been reported to prevent influenza infection (Takahashi and Kozawa, 2021). Therefore, we considered that PHGG may be able to attenuate the symptoms caused by SARS-CoV-2 infection as well. The aim of this study is to examine the effects of PHGG in preventing bodily deterioration caused by SARS-CoV-2 infection.

## Materials and methods

### Animal experiment

SARS-CoV-2 infects humans through ACE2 expressed in the lungs, blood vessels, and intestinal epithelia (Hikmet et al., 2020). The virus has a low affinity to mouse ACE2, so it is not highly virulent in mice (Golden et al., 2020; Wan et al., 2020). Meanwhile, SARS-CoV-2 has a high affinity for hamster ACE2 and can cause infection (Chan et al., 2020), so hamsters are commonly used for SARS-CoV-2 infection animal experiments (Imai et al., 2020). This model is able to recapitulate several hallmark features of COVID-19 in humans, such as inflammation in the lungs and changes in the gut microbiota and gut metabolites (Sencio et al., 2022). Four-week-old female Syrian hamsters were purchased from Japan SLC, Inc. PHGG was purchased from Nestle Japan, Ltd. Hamsters were randomly divided into two groups and given a 5% (w/w) PHGG-supplemented AIN-93G diet (CLEA Japan, Inc.), 5% (w/w) corn starch was replaced with 5% (w/w) PHGG, or AIN-93G control diet for two weeks. SARS-CoV-2 exposure was performed by intranasal application of viral suspension (150 μL PBS containing 1.5 × 10^6^ pfu of an ancestral SARS-CoV-2 strain) to hamsters under anaesthesia. Hamsters were considered to have reached the endpoint at 70% of starting weight. A different set of hamsters was used for gut microbiome and metabolite analysis. After two weeks of PHGG supplementation, the faeces and serum of each group were collected, and the microbiome profile and metabolites were analyzed. All experiments were performed in enhanced biosafety level 3 (BLS-3) containment laboratories at the University of Tokyo, in accordance with the institutional biosafety operating procedures. All animal experiments were performed in accordance with the University of Tokyo’s Regulations for Animal Care and Use, which were approved by the Animal Experiment Committee of the Institute of Medical Science, the University of Tokyo (PA15–92, PA19–87, PA22–33).

### DNA extraction

The DNA of faecal samples was extracted according to the following steps. First, faecal samples were lyophilized by a VD-800R lyophilizer (TAITEC) for at least 18 hours. Freeze-dried faeces (10 mg) were suspended in 300 μl of 10% (w/v) SDS/TE (10 mM Tris–HCl, 1 mM EDTA, and pH 8.0) solution and 300 μl of phenol/chloroform/isoamyl alcohol (25:24:1, Nakalai Tesque). Then, the mixture was homogenized with 3.0 mm and 0.1 mm zirconia beads by ShakeMaster® NEO homogenizer (Biomedical Science, Tokyo, Japan) for 15 min at 1,500 × g. After that, samples were centrifuged for 10 min at max speed and 200 μl of the aqueous phase was used for DNA extraction. DNA extraction was performed by the automated DNA Extraction system GENE PREP STAR PI-480 (Kurabo Industries Ltd.) according to the manufacturer’s protocol.

### Gut microbiome analysis

The gut microbiome profile of stool samples was analyzed by 16S rRNA amplicon sequencing with the following procedure. The V1-V2 variable region of stool DNA was amplified by universal primer set 27F-mod (5′-AGRGTTTGATYMTGGCTCAG-3′) and 338R (5′-TGCTGCCTCCCGTAGGAGT-3′) using Gflex DNA polymerase (Takara) (Yang et al., 2019). After that, the amplified DNA products were sequenced by the next-generation sequencer Miseq (Illumina). The gut microbiome profile was analyzed by Qiime2 (version 2021.11). Sequence data were trimmed and processed by using the DADA2 pipeline for quality filtering and denoising (options: –p-trunc-len-f 285 –p-trunc-len-r 215). The denoised sequences were assigned to taxa using the Silva SSU Ref Nr 99 (version 138) database with the “qiime feature-classifier classify-sklearn” command with default parameters. Unifrac distance was calculated using 12,212 reads per sample with “qiime diversity core-metrics-phylogenetic” command.

### Measurement of SCFAs and organic acids

Faecal samples were lyophilized by a VD-800R lyophilizer (TAITEC) for at least 18 hours. Freeze-dried faeces were homogenized with 3.0 mm zirconia beads by ShakeMaster® NEO homogenizer (Biomedical Science, Tokyo, Japan) for 10 min at 1,500 × g. 10 mg of faecal samples were used for the analysis. Measurement of SCFAs (formate, acetate, propionate, isobutyrate, butyrate, isovalerate, and valerate) and organic acids (lactate and succinate) was performed by 7890 series gas chromatography-mass spectrometry (GC–MS, Agilent Technologies, CA, USA) using previously described methods (Hashimoto et al., 2023).

### Measurement of bile acids

Faecal samples were lyophilized by a VD-800R lyophilizer (TAITEC) for at least 18 hours. Freeze-dried faeces were homogenized with 3.0 mm zirconia beads by ShakeMaster® NEO homogenizer (Biomedical Science, Tokyo, Japan) for 10 min at 1,500 × g, and 10 mg of faecal sample or 50 μl of serum sample were used for the analyses. Measurement of bile acids was performed by 1260 Infinity II liquid chromatography-mass spectrometry (LC–MS, Agilent Technologies, CA, USA) using previously described methods (Hashimoto et al., 2023).

### Statistical analyses

Statistical analyses and correlation analysis (method: Spearman) were performed using R (version 4.2.2). The survival rate was compared using the survival package of R and the generalized Wilcoxon test. The Wilcoxon-Rank-Sum test was used to compare the other data of the two groups.

## Results

### PHGG diet attenuated SARS-CoV-2 infection

**Figure 1.**
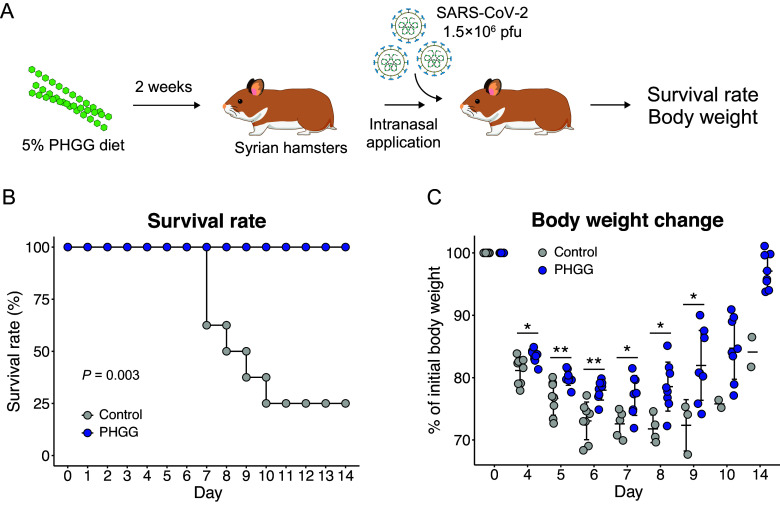
**PHGG diet improved the survival rate and body weight reduction caused by SARS-CoV-2 infection in Syrian hamsters.** (A) Experimental procedures of SARS-CoV-2 infection experiment using Syrian hamsters are shown, (B) Survival rates of hamsters after exposure to SARS-CoV-2. **, *P* < 0.01 (generalized Wilcoxon test), and (C) Body weight change of hamsters after exposure to SARS-CoV-2. *, *P* < 0.05 **, *P* < 0.01 (Wilcoxon rank sum test).

We used Syrian hamsters to assess the preventative effects of PHGG on SARS-CoV-2 infection. Two weeks after exposure to SARS-CoV-2, all hamsters in the PHGG group survived SARS-CoV-2 infection while the control group only showed a 25% survival rate (*P* = 0.003) (Figure 1AB). PHGG also significantly attenuated body weight loss during the measurement period (Figure 1C). By Day 7, the mean body weight was 77.1% of the initial weight in the PHGG group, while in the control group, the mean body weight was 72.6% (P < 0.05, Figure. 1C). Moreover, compared to the control group, the PHGG group more rapidly recovered their body weight (Figure 1C). By Day 9, the mean body weight recovered to 82.0% of the initial weight in the PHGG group, while the control group’s mean body weight remained at 72.6% (P < 0.05, Figure 1C).

### PHGG diet altered the gut microbiome profile

**Figure 2.**
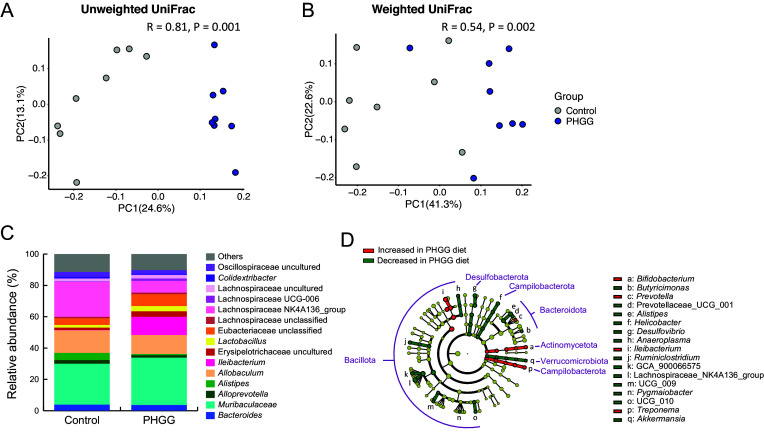
**PHGG diet altered the gut microbiome profile of Syrian hamsters.** (A) Unweighted, (B) weighted UniFrac analyses and ANOSIM of the gut microbiome profile in both control and PHGG groups, (C) Relative abundance of the gut microbiome, and (D) LEfSe analysis of the gut microbiome profile.

**Figure 3.**
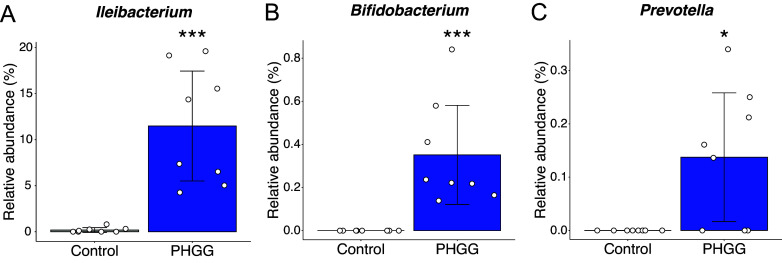
**SCFA producers increased in the PHGG group.** Relative abundance of (A) *Ileibacterium*, (B) *Bifidobacterium*, and (C) *Prevotella.* *, *P* < 0.05 ***, *P* < 0.001 (Wilcoxon rank sum test).

We assumed that the attenuation of SARS-CoV-2 pathogenicity by PHGG was related to changes in the gut microbiome since PHGG was reported to change the gut microbiome profile (Ohashi et al., 2015). Therefore, to elucidate the mechanism of this inflammation alleviatory effect, we performed gut microbiome analysis on a different set of hamsters that were given a 5% PHGG-supplemented diet or a control diet for two weeks without the exposure to SARS-CoV-2. In unweighted UniFrac analysis, the R-value of analysis of similarity (ANOSIM) was 0.81 (P = 0.001), while the R-value of ANOSIM was 0.54 (P = 0.002) in weighted UniFrac analysis (Figure 2AB). These data showed that the PHGG diet significantly altered the gut microbiome profile of hamsters. The bacterial composition and LEfSe data indicate that there were distinct microbiome profiles between the two groups (Figure 2CD). The relative abundance of *Ileibacterium*, *Bifidobacterium*, and *Prevotella* genera was significantly increased in the PHGG group, while *Alistipes* and *Desulfovibrio* were significantly decreased (Figure 2CD, Figure 3). Notably, the relative abundance of *Ileibacterium* was increased to 11.48% in PHGG group compared to 0.2% in control group (*P* < 0.001), and *Bifidobacterium* and *Prevotella* were increased to 0.35% and 0.14%, respectively, in the PHGG group while being undetected in the control group (*P* < 0.001 and *P* < 0.05) (Figure 3). This data suggests that the PHGG diet altered the gut microbiome profile of hamsters.

### PHGG diet altered the intestinal metabolome profile

**Figure 4.**
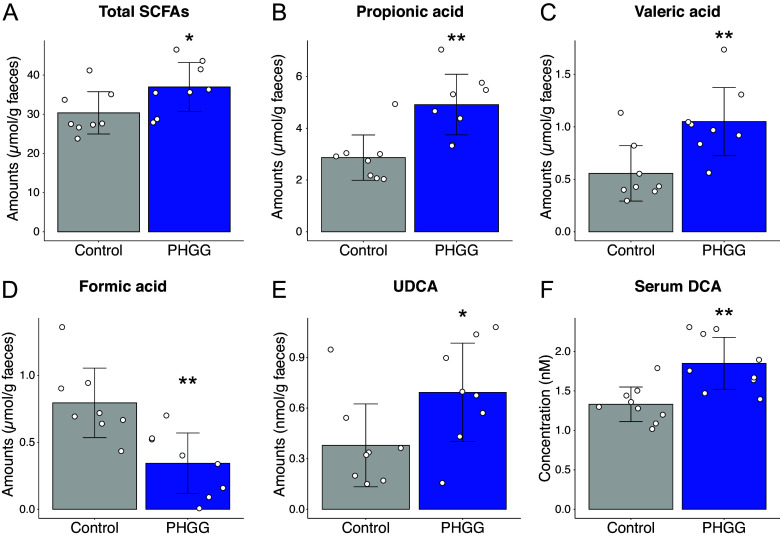
**PHGG diet altered SCFAs and secondary bile acids profiles in the faeces of hamsters.** Comparisons of SCFA concentrations in faeces altered in the PHGG group: (A) Total SCFAs, (B) Propionate, (C) Valerate, (D) Formate, (E) Comparison of UDCA in faeces, and (F) Comparison of DCA in serum. *, *P* < 0.05 **, *P* < 0.01 (Wilcoxon rank sum test).

**Figure 5.**
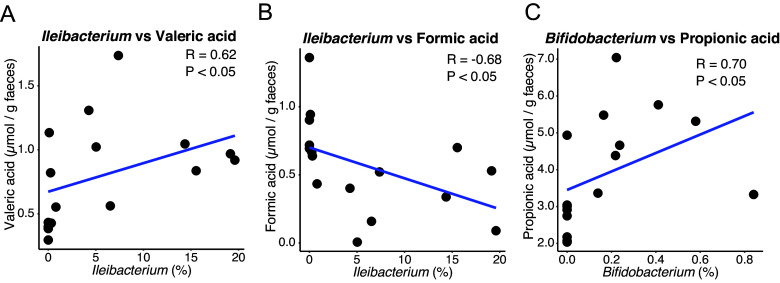
**Gut microbes correlated with the amount of SCFAs.** Correlation analysis between *Ileibacterium* and (A) valerate or (B) formate, and (C) *Bifidobacterium* with propionate.

Since *Bifidobacterium* and *Prevotella* are known SCFA producers, we performed metabolome analysis to investigate SCFA and bile acid production in the gut environment. According to the results, the total amount of SCFAs was significantly increased to 36953.6 ± 6220.8 (nmol/g faeces) in the PHGG group compared to 30352.4 ± 5394.8 (nmol/g faeces) in the control group (*P* < 0.05) (Figure 4A, Supplementary Table S1). Specifically, propionic acid and valeric acid levels were significantly increased in the PHGG group (4916.5 ± 1171.9 and 1049.8 ± 325 nmol/g faeces) compared to the control group (2864.9 ± 876.8 and 556 ± 263.9 nmol/g faeces, *P* < 0.01 respectively) (Figure 4BC, Supplementary Table S1), while formic acid levels were decreased in the PHGG group (343.4 ± 226 nmol/g faeces) compared to the control group (794.6 ± 259.9 nmol/g faeces, *P* < 0.01) (Figure 4D, Supplementary Table S1). We also analyzed the primary and secondary bile acid profile in the faeces, as they have been reported to show anti-inflammatory effects and thus the potential to attenuate the severity of COVID-19 (Fan and Pedersen, 2021; Wang et al., 2022). According to the results, the amounts of anti-inflammatory secondary bile acid UDCA were found to be increased to 0.69 ± 0.29 (nmol/g faeces) in the faeces of PHGG-group hamsters compared to 0.38 ± 0.25 (nmol/g faeces, *P* < 0.05) in the control group (Figure 4E, Supplementary Table S2). Besides, deoxycholic acid (DCA), which has been observed to confer anti-infective effects against SARS-CoV-2 (Ichinohe et al., 2022), was significantly higher in the serum of PHGG group hamsters (1848.5 ± 329.4 nmol/g) compared to the control group (1329.9 ± 219 nmol/g, *P* < 0.01) (Figure 4F, Supplementary Table S3). Next, we attempted to link these changes in the intestinal metabolite composition to compositional changes in the gut microbiota that could be associated with the patterns we observed. We performed correlation analysis of *Ileibacterium*, *Bifidobacterium*, and *Prevotella*, the three genera that showed statistically significant increases in PHGG-group hamsters, against SCFAs and secondary bile acids in faeces and serum. As a result, we found that *Ileibacterium* was positively correlated with valeric acid (R = 0.62, P < 0.05), while it was negatively correlated with formic acid (R = − 0.68, P < 0.05) (Figure 5AB). Interestingly, *Bifidobacterium* showed a positive correlation with propionic acid (R = 0.70, P < 0.05), though propionic acid production ability by *Bifidobacterium* has not been reported in the literature (Figure 5C).

## Discussion

Our data indicate that PHGG administration significantly suppressed morbidity and mortality in SARS-CoV-2-infected Syrian hamsters through possible effects on attenuating inflammation, though this remains to be confirmed in future studies. Furthermore, our results show that PHGG administration altered the gut microbiome profile and metabolites, suggesting a mechanistic link to the antiviral effects. Studies have suggested that dietary intervention may increase resistance to SARS-CoV-2 infection through modulation of the gut microbiota and its associated metabolites (Zhang et al., 2021; Gutiérrez-Castrellón et al., 2022; Zhang et al., 2022b), which is further supported by our study results. That is, modulation of the gut microbiota by dietary fibre may contribute to positive outcomes in response to SARS-CoV-2 infection.

In our study, relative abundances of *Ileibacterium*, *Bifidobacterium*, and *Prevotella* were significantly increased in the PHGG diet group (Figure 3). *Ileibacterium* are known to be associated with polysaccharide metabolism due to their increased abundance in manno-oligosaccharide-gavaged mice, and they also appear to possess the key butyrate pathway genes *atoA/D* genes according to metagenomic sequence data (Wang et al., 2021; Cabral et al., 2022), implying that they are able to produce SCFAs. Thus, it is possible that PHGG increases the abundance of bacteria such as *Ileibacterium*, resulting in a higher amount of SCFAs in the intestines. As a matter of fact, the total amount of SCFAs was increased in the PHGG diet group in our study (Figure 4A), supporting this hypothesis. In several human clinical studies, the relative abundance of *Bifidobacterium* was increased in those consuming a PHGG-supplemented diet (Okubo et al., 1994; Ohashi et al., 2015). Depletion of *Bifidobacterium* in COVID-19 patients has been reported in multiple cases and is inversely associated with disease severity (Al Bataineh et al., 2021; Reinold et al., 2021; Hazan et al., 2022; Taufer and Rampelotto, 2023). In fact, a clinical study showed that an oral booster of *Bifidobacterium* significantly lowered the blood IL-6 levels of administered patients and reduced the length of hospital stay (Bozkurt and Bilen, 2021). *Prevotella* was also reported to be increased with dietary PHGG supplementation in humans (Abe et al., 2023) and was similarly depleted in COVID-19 patient stool samples in several clinical studies (Al Bataineh et al., 2021; Gaibani et al., 2021). These results suggest the existence of a relationship between the aforementioned bacteria and SARS-CoV-2 infection, and we thus hypothesize that the increase of certain members such as *Ileibacterium*, *Bifidobacterium*, and *Prevotella* in the PHGG diet group could be related to the positive outcomes observed in the study.

Administration of the PHGG-supplemented diet also increased SCFA concentrations in hamsters (Figure 4A), similar to human study results (Ohashi et al., 2015). Valeric acid and propionic acid, which were increased in the PHGG group, are known for their anti-inflammation effects (Tedelind et al., 2007; Li et al., 2020). Oral gavage of valeric acid suppressed the pro-inflammatory cytokines interleukin-6 (IL-6) and tumor necrosis factor-alpha (TNFα) levels in peripheral blood, and restored the gastrointestinal tract function and intestinal epithelial integrity in mice exposed to radiation (Li et al., 2020). In *in vitro* experiments, propionate decreased LPS-induced TNFα production by neutrophils and IL-6 production in inflamed colon organ cultures derived from mice (Tedelind et al., 2007). Virus infection would lead to the polarization of pro-inflammatory M1 phenotype macrophages, which may cause cytokine storms (Jardou and Lawson, 2021). It has been proposed that SCFAs could be used to alleviate immune system overactivation in COVID-19 (Jardou and Lawson, 2021). Propionic acid has been shown to suppress mice dextran sulfate sodium-induced colitis and reduce M1-phenotype macrophage polarization through inhibition of the MAPK signalling pathway in an in vitro experiment (Wu et al., 2023). Therefore, the increases in valeric acid and propionic acid might contribute to the alleviation of the inflammation caused by SARS-CoV-2 infection. In our study, valeric acid was positively correlated with *Ileibacterium* abundance (Figure 5A). As mentioned above, although *Ileibacterium* is known for its association with SCFA metabolism (Wang et al., 2021; Cabral et al., 2022), it has not been reported to produce valeric acid. Therefore, further investigation into this relationship is necessary. Propionic acid, which is well-known for its ability to enrich *Bifidobacterium* and is often used for its isolation (Beerens, 1991), was positively correlated with *Bifidobacterium* abundance. On the other hand, *Bifidobacterium* itself has not been reported to produce propionic acid. It is likely that the increase in propionic acid was due to the metabolism of other bacteria. *Prevotella*, whose relative abundance was increased in the PHGG group, has been reported for its capability to produce propionic acid (Strobel, 1992; Zhang et al., 2023). Accordingly, we speculate that *Prevotella* might contribute to the increase of propionic acid in the PHGG group. Finally, formic acid, which has been reported to be elevated in inflammation-associated dysbiosis (Hughes et al., 2017), was also decreased in the PHGG diet group. Taken together, the observed increases in anti-inflammatory SCFAs valeric acid and propionic acid and a decrease in inflammation-associated SCFA formic acid appear to be associated with the improved disease outcomes seen in PHGG-fed hamsters. We hypothesize that the modulation of SCFAs is one of the mechanisms by which SARS-CoV-2 infection was attenuated.

Gut microbes convert primary bile acids to various secondary bile acids (Winston and Theriot, 2020). The PHGG-supplemented diet used in our study increased the amount of the secondary bile acid UDCA in hamster faeces. Additionally, DCA was increased in the serum of the PHGG-fed group. A previous study showed that UDCA works as an antagonist of FXR and reduces ACE2 expression in conjunction with SARS-CoV-2 infection symptoms in Syrian hamsters (Brevini et al., 2023). Additionally, in two different cohort studies, people who received UDCA treatment for chronic liver disease or after a liver transplant had better clinical outcomes after developing COVID-19. In addition, another FXR ligand, DCA, has been reported to have anti-infective effects against SARS-CoV-2 in Syrian hamsters (Nagai et al., 2023). In our study, faecal UDCA and serum DCA were increased in Syrian hamsters after PHGG consumption (Figure 4E, F). Accordingly, we hypothesize that PHGG consumption enhances resistance to SARS-CoV-2 infection through FXR binding, as activation of this receptor is found to reduce the expression of ACE2 as mentioned previously (Brevini et al., 2023).

Several studies have suggested that probiotic bacteria such as *Bifidobacterium* and *Lactobacillus* may improve the clinical outcome of SARS-CoV-2 infection (Ceccarelli et al., 2020; Zhang et al., 2021). A clinical trial using encapsulated synbiotic formula SIM01 consisting of 3 *Bifidobacterium* strains and 3 prebiotic polysaccharides showed that SIM01 received COVID-19 patients increased the SARS-CoV-2 immunoglobulin G antibody and reduced the pro-inflammatory immune markers, which suggested that SIM01 has the potential to increase resistance to SARS-CoV-2 infection (Zhang et al., 2022b). Compared to probiotic bacteria, the production cost of prebiotic dietary fibre PHGG is lower, and it is also easier to manage logistically. Thus, PHGG may be more accessible for use in daily life, but further studies are first needed to clarify the effects of PHGG on preventing SARS-CoV-2 infection in human clinical trials.

Conclusively, our study indicates that PHGG supplementation increases the survival rate, attenuates body weight loss, and promotes recovery in SARS-CoV-2-infected hamsters. Using a different set of non-infected hamsters, we showed PHGG modulated the gut microbiome and increased valeric acid and propionic acid, as well as UDCA, in faeces, and DCA in serum. Further study is required to measure the gut microbiome and gut metabolite outcomes within a single SARS-COV-2-infected group of hamsters to confirm this connection. It is considered that PHGG supplementation has the potential to prevent bodily deterioration caused by COVID-19 by repressing the inflammation response and preventing the severe symptoms caused by cytokine storms. Further analysis of inflammatory cytokines and other markers would provide mechanistic insight. Nevertheless, dietary consumption of PHGG is a simple and easily accessible intervention. To demonstrate the benefit of PHGG in SARS-CoV-2 infection and its importance in public health, human clinical trials are necessary. Further human trials of PHGG to investigate a therapeutic role in SARS-CoV-2 infection are required as well.

## Supporting information

Yang et al. supplementary material Yang et al. supplementary material

## Data Availability

The microbiome analysis data have been deposited in the DNA Data Bank of Japan (DDBJ) Sequence Read Archive (http://trace.ddbj.nig.ac.jp/dra/) as DRA016419.

## Supplementary material

The supplementary material for this article can be found at http://doi.org/10.1017/gmb.2024.7.
