# Partially hydrolyzed guar gum attenuates symptoms and modulates the gut microbiota in a model of SARS-CoV-2 infection – CORRIGENDUM

**DOI:** 10.1017/gmb.2026.10030

**Published:** 2026-06-16

**Authors:** Jiayue Yang, Isaiah Song, Misa Saito, Tenagy Hartanto, Takeshi Ichinohe, Shinji Fukuda

The authors apologise for the error in the concentration described in the “Materials and Methods” section.

The text reads, “Freeze-dried faeces (10 mg) were suspended in 300 μl of 10% (w/v) SDS/TE (10 mM Tris–HCl, 1 mM EDTA, and pH 8.0) solution and 300 μl of phenol/chloroform/isoamyl alcohol (25:24:1, Nakalai Tesque).” The text should read, “Freeze-dried faeces (10 mg) were suspended in 300 μl of 1% (w/v) SDS/TE (10 mM Tris–HCl, 1 mM EDTA, and pH 8.0) solution and 300 μl of phenol/chloroform/isoamyl alcohol (25:24:1, Nakalai Tesque).”

The authors apologise for this error and wish to correct them via this notice.
