# Supplementary material for: Partially hydrolyzed guar gum attenuates symptoms and modulates the gut microbiota in a model of SARS-CoV-2 infection
Source: Gut Microbiome (Camb). 2025 Jan 14;6:e1. doi: 10.1017/gmb.2024.7 (PMC11810603; doi:10.1017/gmb.2024.7)
Supplement: Yang et al. supplementary material [file S2632289724000070sup001.pdf]

Supplementary Table 1. Amounts of SCFAs and organic acids in faeces.

SCFAs and organic acids	Control (nmol / g faeces) Average ± Standard deviation	PHGG (nmol / g faeces) Average ± Standard deviation	*P* value
Acetic acid	22326.6 ± 3794.6	25358.5 ± 4016.5	N.S.
Butyric acid	3552.0 ± 3159.8	5069.7 ± 1896.2	N.S.
Propionic acid	2864.9 ± 876.8	4916.5 ± 1171.9	**
Formic acid	794.6 ± 259.9	343.4 ± 226.0	**
Valeric acid	556 ± 263.9	1049.8 ± 325.0	**
Isobutyric acid	160.8 ± 51.0	139.8 ± 66.9	N.S.
Isovaleric acid	97.4 ± 41.6	75.9 ± 34.1	N.S.
Total SCFAs	30352.4 ± 5394.8	36953.6 ± 6220.8	*
Succinic acid	278.7 ± 166.7	386.3 ± 261.4	N.S.
Lactic acid	188.1 ± 279. 0	21.6 ± 20.8	N.S.

Average values ± standard deviation for each SCFA (and organic acid) and group are shown. Comparisons of the two groups were performed by Wilcoxon Rank Sum test and *P* values are shown on the right. *, $P < 0.05$ **, $P < 0.01$, N.S., no significant differences.

Supplementary Table 2. Amounts of bile acids in faeces.

Bile acids	Control (nmol / g faeces) Average ± Standard deviation	PHGG (nmol / g faeces) Average ± Standard deviation	*P* value
DCA	81.75 ± 40.88	82.62 ± 15.81	N.S.
LCA	78.44 ± 22.75	92.66 ± 8.92	N.S.
CA	8.25 ± 5.33	5.24 ± 1.74	N.S.
UDCA	0.38 ± 0.25	0.69 ± 0.29	*
a-MCA/ w-MCA	0.29 ± 0.45	0.19 ± 0.11	N.S.
CDCA	0.06 ± 0.14	0.03 ± 0.09	N.S.
TCDCA	0.04 ± 0.04	0.02 ± 0.03	N.S.
b-MCA	0.03 ± 0.09	0.00 ± 0.00	N.S.
GDCA	0.02 ± 0.03	0.02 ± 0.03	N.S.
TLCA	0.01 ± 0.02	0.03 ± 0.05	N.S.
TDCA	0.01 ± 0.03	0.02 ± 0.05	N.S.

Average values ± standard deviation for each faecal bile acid and group are shown. Comparisons of the two groups were performed by Wilcoxon Rank Sum test and *P* values are shown on the right. *, $P < 0.05$, N.S., no significant differences.

Supplementary Table 3. Concentration of bile acids in serum.

Bile acids	Control (nM) Average ± Standard deviation	PHGG (nM) Average ± Standard deviation	*P* value
CA	15220.3 ± 4298.1	16868.7 ± 4531.1	N.S.
DCA	1329.9 ± 219.0	1848.5 ± 329.4	**
GCDCA	39.2 ± 110.9	157.2 ± 261.1	N.S.
GDCA	N.D.	57.8 ± 163.5	N.S.
GCA	N.D.	183.4 ± 518.8	N.S.

Average values ± standard deviation for each serum bile acid and group are shown. Comparisons of the two groups were performed by Wilcoxon Rank Sum test and *P* values are shown on the right. **, $P < 0.01$, N.S., no significant differences.
